# Single infection with *Batrachochytrium dendrobatidis* or *Ranavirus* does not increase probability of co-infection in a montane community of amphibians

**DOI:** 10.1038/s41598-020-78196-3

**Published:** 2020-12-03

**Authors:** Jaime Bosch, Camino Monsalve-Carcaño, Stephen J. Price, Jon Bielby

**Affiliations:** 1 Research Unit of Biodiversity (CSIC, UO, PA), Oviedo University - Campus Mieres, 33600 Mieres, Spain; 2 Museo Nacional de Ciencias Naturales-CSIC, José Gutiérrez Abascal 2, 28006 Madrid, Spain; 3 UCL Genetics Institute, Darwin Building, Gower Street, London, WC1E 6BT UK; 4 Institute of Zoology, Zoological Society of London, Regents Park, London, NW1 4RY UK; 5 School of Natural Sciences and Psychology, Liverpool John Moores University, James Parsons Building, Byrom Street, Liverpool, L3 3AF UK

**Keywords:** Herpetology, Conservation biology, Viral infection

## Abstract

Understanding the occurrence and consequence of co-infections can be useful in designing disease management interventions. Amphibians are the most highly threatened vertebrates, and emerging pathogens are a serious threat to their conservation. The amphibian chytrid fungus and the viruses of the *Ranavirus* genus are already widely distributed, causing disease outbreaks and population declines worldwide. However, we lack information about the occurrence and consequences of coinfection with these pathogens across age-classes of amphibian hosts. Here, we analyze the occurrence of infection of the amphibian chytrid fungus and ranaviruses during one season in two susceptible amphibian species at two different locations at which outbreaks have occurred. We found that the co-occurrence of both pathogens in a particular host is not common except in highly susceptible life-stages, and that single infections are the most common situation. Moreover, we found that the occurrence of one pathogen in a particular host did not predict the occurrence of the other. We attribute these results to the niches in which both pathogens proliferate in amphibian hosts.

## Introduction

The majority of hosts carry a number of infectious parasites and pathogens (hereafter pathogens) within their body, meaning that co-infections within a host represent the most common context of host–pathogen dynamics^1^. The basic ecological assumption is that pathogens infecting the same host have the potential to affect each other in different main ways. Antagonism can occur where one pathogen has a negative effect on the development of the other, either via trophic interactions or cross-immunity, which would result in a negative relationship between infection levels of different pathogens. On the other hand, pathogens may exhibit synergism, where one pathogen promotes the development of the other, perhaps by diverting the immune response of the host or suppressing further immune response, which could lead to a positive relationship between infection levels of the pathogens in question^1^. However, studies of co-infection in natural systems can be affected by many confounding factors and require cautious interpretation^2^. If data come from single sample surveys of natural populations or from controlled-infection studies in the laboratory, there are some considerable limitations that may hinder the generality of their findings^3^.

While field data typically show that most individuals harbor multiple pathogens, short-term surveys may limit our predictive and explanatory power because they lack detail on the effects of seasonality and community structure in the host–pathogen system. Similarly, infection studies in the laboratory typically involve infecting hosts with controlled doses of two or more parasites either simultaneously or sequentially, and can frequently yield unrepeatable results^4^. For example, the experimental conditions may not realistically represent the operational ranges of either the hosts or the pathogens in question. It is not always clear how captive or laboratory conditions affect either the infection probability of the host as their immune response may be affected^5–7^Further, the infectiousness of the pathogen may be compromised due to fitness costs experienced in laboratory culturing conditions^8,9^. Another example is that laboratory infection studies typically use high doses of a pathogen to ensure infection success. We know that the response of hosts is often dose-dependent, and that the scale of these laboratory doses are unlikely to reflect the doses an individual will face in nature (e.g.^10,11^). As such, although experimental challenge experiments provide important information on host–pathogen dynamics, they often represent a worst-case scenario as experiments in the laboratory are often conducted in conditions that are ideal for the pathogens at very high doseage. It istherefore hard to know how to extrapolate from these findings to the occurrence and consequences of coinfection in natural systems.

In recent decades emerging infectious diseases have been implicated in the decline of amphibians around the world^12–14^. Three high-profile pathogens associated with these declines are the chytrid fungi *Batrachochytrium dendrobatidis* (*Bd*) and *B. salamandrivorans*, causal agents of chytridiomycosis, and iridovirids in the genus *Ranavirus* (*Rv*), causal agents of ranavirosis. The majority of research on these pathogens is limited to single infections, yet undetected co-infections may be an unrecognized threat to amphibian species. While the co-occurrence of and coinfection with these pathogens could represent a serious conservation issue for some species, little information is currently available on whether they commonly co-occur geographically, whether they co-infect hosts, or what are their possible synergetic effects are within a host.

In terms of their ecology, infection progression, and impacts on hosts, both *Rv* and *Bd* are water-borne parasites that are often associated with infections in the larval life-stage of amphibians^14,15^. However, while in temperate regions *Bd* often imposes the highest mortality during the meta-morphic and post-metamorphic life-stage of amphibians (e.g. in species of the genus *Alytes*), *Rv* more frequently imposes mortality on every life-stage including larvae. More importantly, while ranavirosis incidents in wild populations can be more frequent and more severe at higher temperatures^14^, *Bd* infections are more intense at low temperatures^16^, which may suggest that the two pathogens have different optimal temperatures, and that host–pathogen dynamics may vary greatly between these pathogens even for the same host species.

The Picos de Europa National Park (PNPE) is a protected area in the north of Spain that has experienced ranavirosis-related population declines affecting some of its nine species of amphibians. Outbreaks of ranavirosis were first observed in the park in 2005 and resulted in severe and dramatic declines at several locations, while *Bd* has also been present in the region since at least 2005 without observations of chytridiomycosis^12^. The common midwife toad (*Alytes obstetricans,* hereafter *Ao*) and the alpine newt (*Ichthyosaura alpestris*, hereafter *Ia*) are the most common species in the park and remain the most easily sampled and monitored despite the severe impact of ranavirus. These two species are frequently infected with these pathogens, and are responsible for their maintenance and their spread^17–19^. *Ao* is a species that carries *Bd* infection at high prevalence, and is severely affected by the associated disease, chytridiomycosis^17,20^. *Ia* has been associated with the spread and maintenance of *Rv* as both a native host and as an invasive carrier of the pathogen^21^. Therefore, the PNPE and its amphibian community provide an ideal system to study co-infection with these amphibian pathogens.

Our goal is to assess within-host associations between these two pathogens. We ask the question: is infection with *Bd* associated with infection with *Rv*, or vice versa? We use data collected on these species over a year-long field season to address the following questions: (1) What environmental and ecological variables are important in explaining the variation in probability of infection with each of these two pathogens (*Bd* and *Rv*)?, (2) How does the co-infection status of individuals of the two more sensitive hosts vary at two sites with differing size, water temperature, and seasonality?, and (3) Within co-infected hosts, how are the infection loads of these two pathogens linked?

To answer the first question we built two explanatory models examining the probability of infection with each of the two pathogens, *Bd* and *Rv*. These models incorporated individual-level data, including as predictor variables the season, the host species and life-stage, and the infection status with the non-focal pathogen (e.g. for the model explaining the probability of infection with *Bd* we included as a predictor variable the *Rv* infection status of that individual). Based on the apparent differences in optimal temperature between the two host–pathogen combinations, we predict that the infection with one pathogen was not related with the infection of the other one in this study system because of the divergent optima for pathogen infection. To answer the second question we used data on the infection status of individuals of focal species at two focal sites to look for associations between *Bd* and *Rv* infection status and based on previous studies and the thermal optima of the pathogens predict that there would be little evidence of coinfection between the pathogens. To answer the third question we looked at correlations between infection levels in the two pathogens using data from coinfected hosts, with a prediction that there would be no evidence, or even a negative relationship between the pathogen infection levels.

## Results

### General patterns of infection

**Figure 1:**
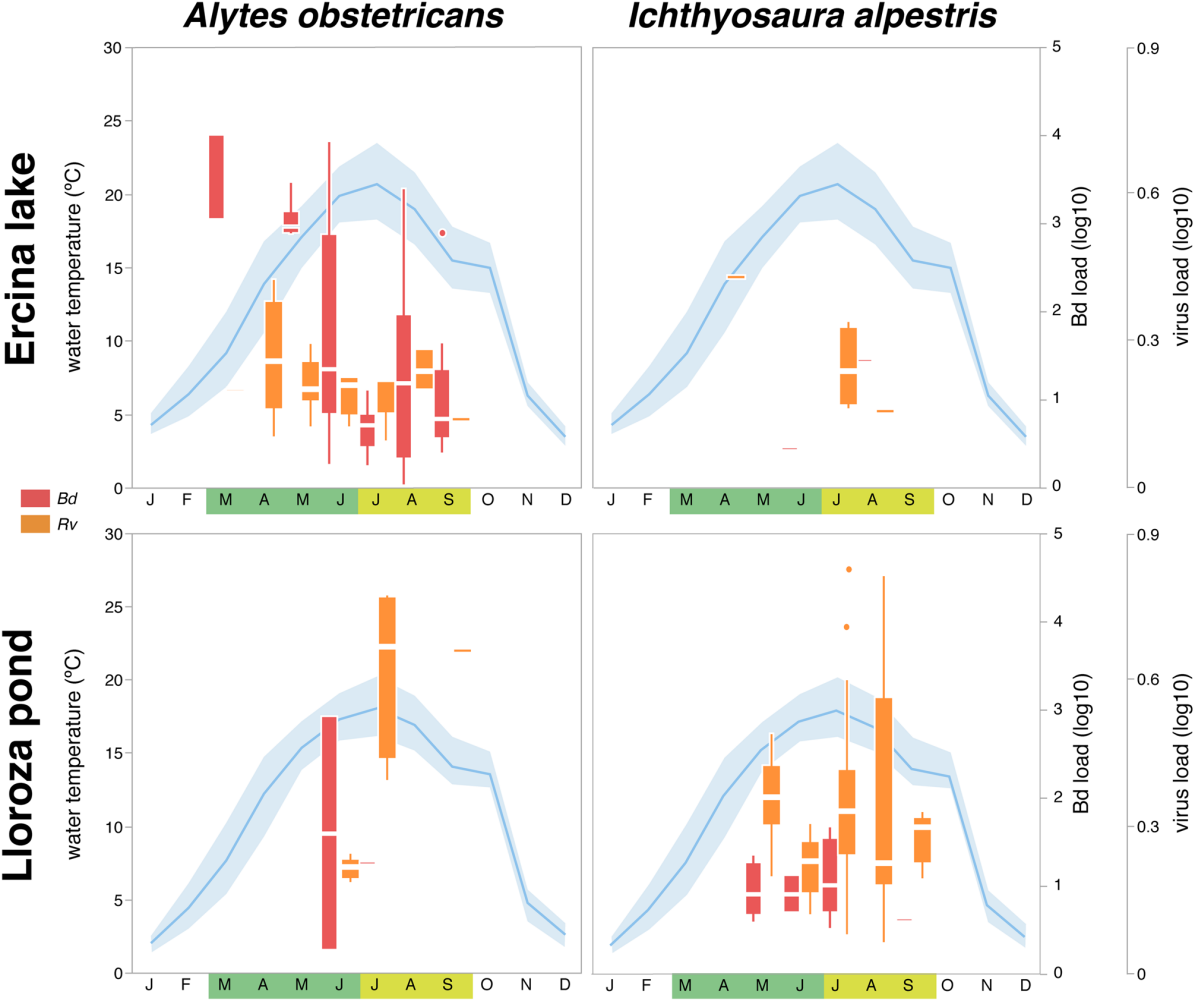
Monthly infection loads (box-plots) for spring months (March–June; green shadow) and summer months (July–September; yellow shadow) for both species sampled (*Alytes obstetricans* and *Ichthyosaura alpestris*) and locations (Ercina lake and Lloroza pond). Infection loads are shown as log transformed genomic equivalents of zoospores for *Bd* (red) and viral genome copies for *Rv* (orange). Horizontal lines depict medians, boxes represent interquartile ranges, whiskers extend to minima-maxima. Water temperatures (maximum, minimum and averaged values; blue lines) are shown for reference.

**Figure 2:**
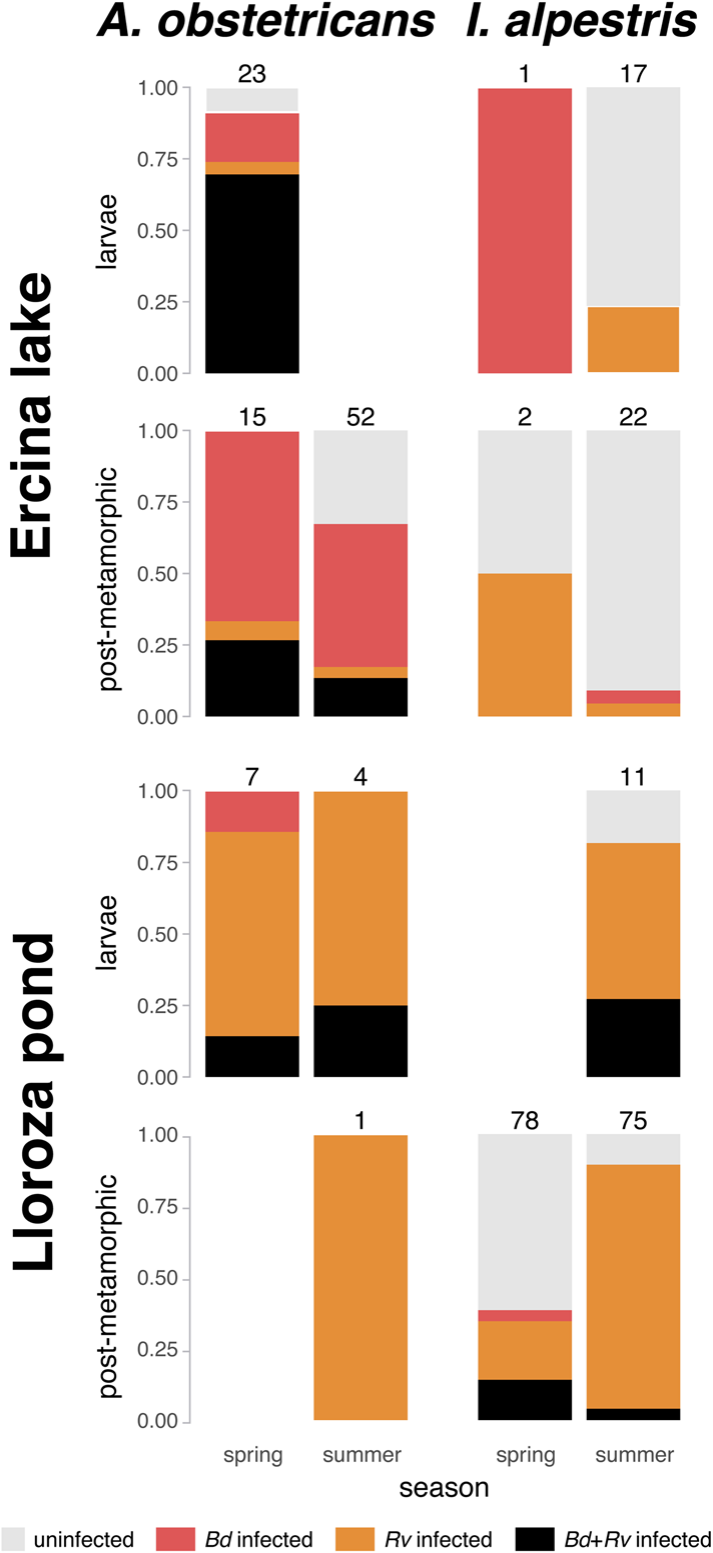
Proportion of uninfected individuals (grey), *Bd* infected individuals (red), *Rv* infected individuals (orange) and *Bd* plus *Rv* co-infected individuals (black) for both sampled species (*Alytes obstetricans* and *Ichthyosaura alpestris*) and locations (Ercina lake and Lloroza pond). Sample sizes appear in numbers.

The infection intensity across species and localities and proportion of infected and coinfected individuals are shown in Figs. 1 and 2. *Ao* consistently exhibited a higher prevalence of infection (with one pathogen or the other or both; Fig. 2) than *Ia*. In *Ao,* most individuals of all life-stages were infected with at least one of the two pathogens. In contrast, most individuals of all *Ia* life-stages were either uninfected or infected with *Rv* and only very rarely with *Bd* (Fig. 2).

A preliminary visual inspection of the data suggests considerable variation in infection for both pathogens across the active season of the amphibian community, suggesting that statistical analyses of our covariates was warranted (Figs. 1 and 2). Analyses via linear models highlighted that infection levels with *Bd* were typically higher during spring than summer, but remained similar across seasons for *Rv*. While *Bd* infection was similar between localities, *Rv* prevalence and infection intensity was higher in Lloroza than in Ercina. Finally, while *Rv* infection did not vary significantly between seasons and life stages, *Bd* infection was higher in larvae than in postmetamorphic individuals.

Eleven *Ao* and 37 *Ia* individuals showed signs of ranavirosis, despite one (9%) and 18 (49%) of these individuals, respectively, testing negative for *Rv* by qPCR. Among positive animals exhibiting signs of ranavirosis, just two out of 12 (17%) *Ao* and three out of 19 (16%) *Ia* individuals tested positive for both *Bd* and *Rv*.

### Analysis of probability of infection

**Figure 3:**
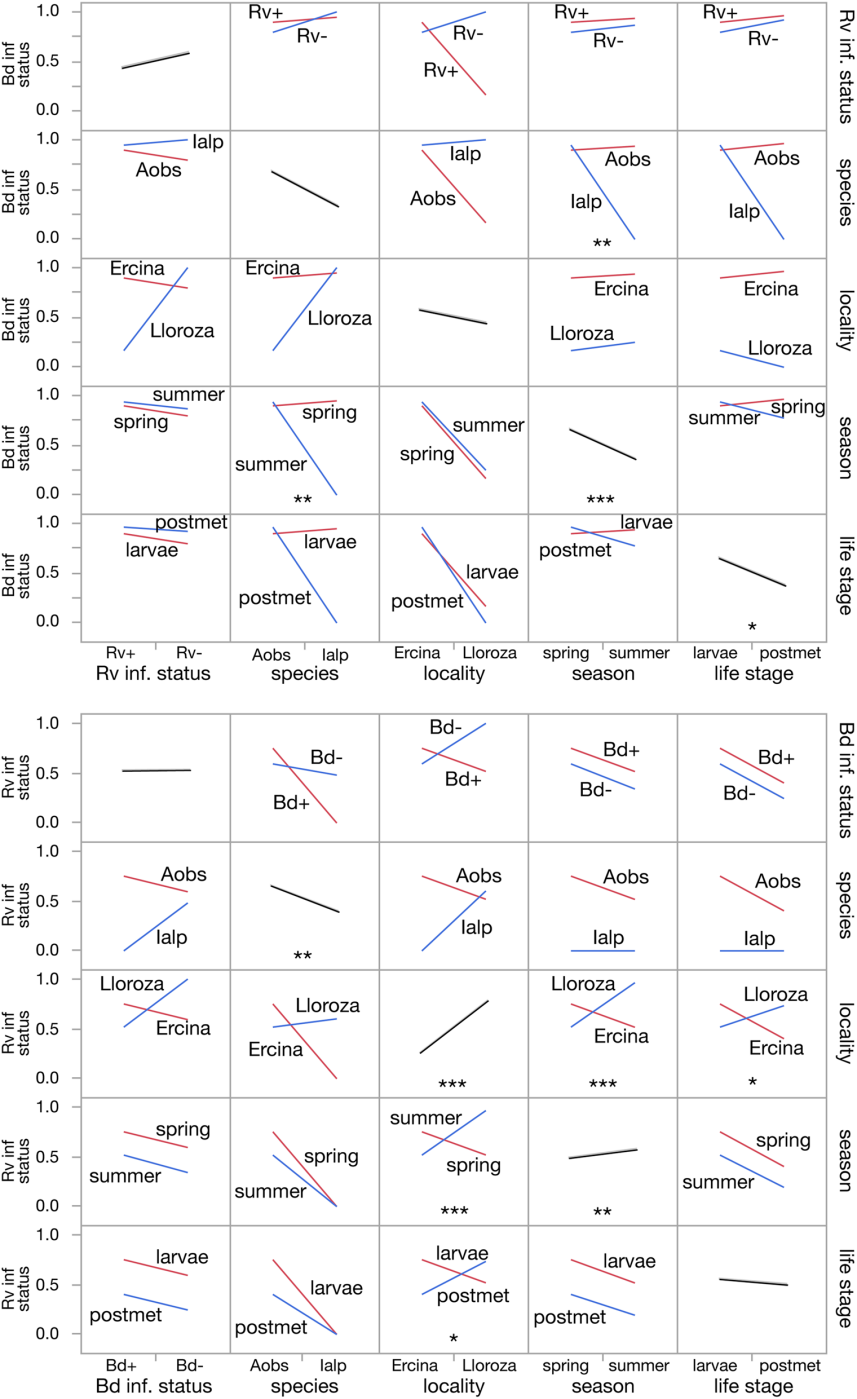
Marginal model plots of the main effects (diagonal) and plots of interaction effects (rest) of the explanatory models for *Bd* and *Rv* infection statuses. For the main effects, the plots show the mean response for each factor value and a 95% confidence band for the simulated means. For the interaction effects the response values predicted by the model are joined by line segments.

The best explanatory model for *Bd* infection status included 14 terms including interactions and explained 45% of the variance, while the best model for *Rv* infection status included 11 terms including interactions and explained 36% (Table 1, Supplementary Table S1). Marginal model plots of the main effects and plots of interaction effects of both explanatory models are shown in Fig. 3. In both cases, the infection status of one pathogen did not, on its own, predict the infection status with the other. The infection status with one pathogen did appear in the best model for both pathogens, but only in interaction with site and species. For more detailed analysis of the coinfection probability at both sites in the focal species, see the results below on the associations between *Rv* and *Bd* infections. On the other hand, other covariates, like species, locality and season, were good predictors of the infection status of individuals for both pathogens, as well many interactions between factors (Fig. 3).

**Table 1:** Best explanatory models for *Bd* and *Rv* infection status, including the significance of whole models, the proportion variance explained (r^2^), and the significant effects for both models sorted by their relative importance.

*Bd* infection status	*Rv* infection status
df = 14, Chi^2^ = 169.3, *p* < 0.0001, r^2^ = 0.45	df = 11, Chi^2^ = 155.1, *p* < 0.0001. r^2^ = 0.36
Season (*p* = 0.0009)	Locality × season (*p* < < 0.0001)
Species × season (*p* = 0.0043)	Locality (*p* < < 0.0001)
*Rv* inf. status × species × locality (*p* = 0.0110)	Season (*p* < 0.0029)
Life stage (*p* = 0.0142)	*Bd* inf. status × species × locality (*p* = 0.0034)
Species × season × life stage (*p* = 0.0302)	Species (*p* = 0.0094)
	Locality × life stage (*p* = 0.0353)

### Associations between Rv and Bd infections

The proportion of uninfected, infected with just one pathogen and coinfected animals across species and localities appear in Table 2. For every combination of species and localities, coinfected animals were never the most abundant, while in three out of four combinations the majority of animals were infected with just one pathogen.

**Table 2:** Number of individuals non infected, infected with just one pathogen (*Bd* or *Rv*), and co-infected with both *Bd* and *Rv* across species and localities, and their statistical differences against the expected values by chance. N = sample size; Aobs, *Alytes obstetricans*; Ialp, *Ichthyosaura alpestris*.

Species	Locality	N	Uninfected	*Bd* or *Rv*	*Bd* and *Rv*	Chi^2^	*p*
Aobs	Ercina	90	19	44	27	10.9	0.0044
Aobs	Lloroza	12	0	10	2	14.0	0.0009
Ialp	Ercina	42	34	8	0	45.1	< 0.0001
Ialp	Lloroza	164	58	89	17	47.7	< 0.0001

Further, when coinfection occurred in a single host, the infection intensities of *Bd* and *Rv* were not significantly correlated and the relationship was negative (n = 46, ρε =  − 0.269, *p* = 0.0537).

### Infection patterns in dead individuals

Six individuals of *Ao* and 30 of *Ia* were found dead during surveys. Two out of six (33%) dead *Ao* tested positive for *Rv* and just one out of six (17%) for *Bd*, but no individuals tested positive for both pathogens. For *Ia*, 28 out of 30 (93%) dead animals tested positive for *Rv*, while just two (7%) were co-infected and none were infected only with *Bd*.

## Discussion

Our findings indicate that, in this system, infection with one pathogen does not necessarily predict infection status with another, even in species that are known to be important hosts for these pathogens. Moreover, our results suggest that these amphibian pathogens showed a negative association, with the presence of one being linked to a lower infection intensity in the other. Together these findings highlight that despite possible synergistic interactions between pathogens^1^ it is not advisable to infer direct negative consequences simply because of the presence of two pathogens. Our data from natural populations indicated that other covariates (locality, season and species) were more important in predicting infection status with a single pathogen; emphasizing the importance of environmental characteristics in host–pathogen dynamics, coinfection patterns and their effects.

The body of evidence suggesting that there is no association in coinfection patterns in these pathogens is growing, giving us a better understanding of amphibian population biology^22,23^. Strong evidence exists that there are no strong synergistic effects on either the prevalence or intensity of infection between *Bd* and *Rv* in either tropical or temperate locations^22,23^. Warne et al*.*^22^ found that there was no link between infection status or intensity with these pathogens in a community of Andean species. Further, these results were consistent across three species that differ in life-history and origin (wild harvested frogs in a live market and free-living captured animals). Similarly, Olori et al*.*^23^ found in a temperate community of amphibians that no such links between infection prevalence or intensity existed in any of 15 species sampled over a 5 year period. Both of these studies were based on New World species, which face similar ecological challenges to those in our study such as high seasonality, albeit with a very different evolutionary lineage within amphibians. Our results suggest that these findings are consistent across phylogeny, with Old and New World species having very similar co-infection dynamics with these pathogens. However, the New World findings are based largely on fully metamorphosed individuals, whereas our study included larval stages, which are known to play a key role in the maintenance and proliferation of infection with both *Bd*^24,25^ and *Rv*^26^. Further, both lethal and sublethal effects of *Bd* on amphibian larvae may occur in that age-class and continue to be incurred into post-metamorphic life^27,28^. The lack of synergism between *Bd* and *Rv* found in larvae here suggests that those costs are unlikely to be exacerbated by the presence of both pathogens.

The lack of cumulative or amplified patterns of infections between these pathogens within a host community also seems to be extended when considering disease emergence and mortality^29^. So, even though we should not underestimate the impact of these pathogens on host populations, as they can lead to severe population declines, the body of evidence at present suggests that co-infections do not promote higher probability of infection, their severity, or their likelihood of disease progression (even when under ideal conditions in the laboratory co-infection can be archived; T.W. Garner, personal communication). Note, however, that contrarily to Bd, actual prevalence of *Rv* can be underestimated in this study because we analyzed toe-clips or tail-clips instead internal organs, when usually liver and internal organs presented higher rates of infection (e.g. von Essen et al.^39^).

The mechanism behind this lack of interaction could provide useful guidance for understanding general aspects of coinfection dynamics. In this particular case, these general aspects may relate to the temperature requirements of the pathogens and the ecology of the hosts. In systems with seasonality like ours, due to the relatively dry terrestrial environment, *Bd* infection is predominantly linked to aquatic life stages (e.g.^25^). Adult *A. obstetricans* are completely terrestrial amphibians and are consistently *Bd* free, while larvae spend up to several years in water and harbour strong infections^25^. This highly aquatic lifestyle could explain why *A. obstetricans* larvae tend to have a high prevalence and strong infection intensity with *Bd* during the winter, but also strong *Rv* infections during the hottest summer weeks. In contrast, adult *I. alpestris* spend much of their time in the water, from April to September, including the end of the summer when *Rv* levels are high, but are more terrestrial during the winter months^19^ when low water temperature may dampen the amphibian immune system^5^. Moreover, the two pathogens studied here have disparate times when infection typically occurs^13,14^, and this association could help to explain why we have detected a limited influence of one pathogen on the other. *Bd* is linked to cooler temperatures and *Rv* to warmer temperatures in montane areas of the northern hemisphere, they probably experience suitable growth conditions only occasionally when one pathogen is decreasing replication and the other is increasing.

Previous research has suggested that exposure to an increasing number of pathogens can affect infection probability and, further, the sequence of infection may be important. For example, Johnson and Hoverman^30^ combined infection data from field-studies with mechanistic experiments looking at interactions among six trematode parasites of amphibians. They found that the increase in parasite richness reduced overall infection success, as predicted by the dilution effect hypothesis. In this case, the sequence of infection mattered, possibly due to priority effects^31^; the effects of parasite richness on host growth and survival varied depending on whether parasites were administered additively or if added species replaced more virulent species. Although our study is based on a smaller number of micro-, rather than macro-parasites, it seems reasonable to suppose that the sequence of infection could be relevant to the relative success of *Bd* and *Rv* in an amphibian host.

While infection with one pathogen does not increase host susceptibility to the second pathogen, that is not to say there are not within-host interactions between them. For example, it is possible that the presence of one pathogen can increase the infection intensity of other infections that are already present within that host, which would otherwise remain at low or moderate infection loads^32,33^. If this type of interaction were to occur within the relatively small number of coinfected hosts, this could increase the likelihood of super-spreading individuals^34,35^, which has important implications for the transmission of infection within these populations and communities.

To summarise, this study outlines that did not find a synergistic relationship between two pathogens of amphibian species. Further, while these pathogens have had serious negative effects on animal welfare and conservation globally^12,15^, and may therefore have been expected to be more severe combined than alone. The specifics of host and pathogen ecology and life history may be one aspect limiting whether pathogens have the opportunity to interact within the host. Similarly, differences in the parts of the host immune response to the pathogen may also help to explain this lack of interaction, as resistance to *Bd* and *Rv* is via different MHC subgroups^36^. Therefore, while pathogen surveillance may identify the presence of multiple pathogens, interactions between them, or increases in their effect may greatly depend on the specifics of the hosts, the pathogens, and the ecological and environmental circumstances in which they occur.

## Methods

The Picos de Europa National Park (PNPE) is a protected wilderness area situated in the north of Spain. The park has a mountainous limestone geology and is used for recreation and stockbreeding, but is also an important wildlife area. Two lakes within the park were the foci of data collection within this study: Ercina lake (1110 m asl and 6.6 ha of maximum surface) and the Lloroza pond (1860 m asl and 3070 m^2^ of surface). These two sites were selected because they host two of the most important populations of the two focal species (*Ao* and *Ia*), and because of the different physical properties of the lakes with associated differences in ecology over the course of the year. Submerged dataloggers (HOBO Pro v2 Water Temperature Logger U22–001, Onset Inc., Bourne, Massachusetts) in each lake provided a continuous half-hourly measurement of water temperature across 1 year.

To obtain data on the infection status of each of these species, all life-stages of both were sampled at these two locations within PNPE throughout their period of activity within the calendar year of 2017. At these high-elevation sites, this period stretches from ice-melt in March/April through to September. For each species: life-stage combination, at each sampling point, as many individuals as possible were sampled up to a maximum of 20 in a given sampling window. For simplicity and to maximize the power of our analyses two life-stages were defined: larval and post-metamorphic. The former included both young of year and over-wintered tadpoles in *Ao*, and the latter combined metamorphic individuals, juveniles and adults.

At both sites, lakeside walking transects were conducted to collect tissue samples for diagnostic tests. Each lake was split into four sections and walked as a transect by a pair of observers at twilight, when amphibian activity is at a maximum. This protocol was conducted to ensure that sufficient data were collected for our analyses.

To sample individuals for diagnostic tests for *Bd* and *Rv*, tissue samples were collected by different means according to the species and life-stage involved. For *Ia* tail-clips were taken from all life-stages, while for *Ao* tail-clips were taken from larval individuals for *Rv* diagnosis and oral swabs for *Bd* diagnostics. Toe-clips were taken from post-metamorphic individuals. All collected tissue samples were fixed in 70% ethanol and swabs were kept dry and refrigerated. Lesions consistent with the clinical signs of ranavirosis (erythema or hemorrhage) in the sampled individuals were noted.

Additionally, dead individuals were collected opportunistically throughout the sampling period. These individuals were important because they could be informative about whether sampling live animals was biased towards those individuals with single infections, or lower levels of infection; if co-infection most commonly resulted in mortality, we might expect dead individuals to be co-infected, potentially with a high infection intensity. Dead individuals were collected and stored in 70% ethanol for qPCR analysis.

DNA was extracted from tissue samples using DNeasy Blood and Tissue Kit (Qiagen, Hilden, Germany) and from swabs with PrepMan Ultra. qPCR for *Bd* and *Rv* was performed in the MNCN-CSIC lab following Boyle et al*.*^37^ for *Bd* and Leung et al*.*^38^ for *Rv* respectively on a MyGo Mini PCR machine. Samples, negative controls and standards with known concentrations of *Bd*/*Rv* were run in duplicate. Samples that showed signs of inhibition (nonsigmoidal amplification) were further diluted to 1:100 (*Bd*) of 1:10 (*Rv*) and re-analyzed. Samples were considered positive when both of the two duplicate analyses revealed infection loads > 0.1 (*Bd*) or > 3 (*Rv*), and the amplification curves have a sigmoidal shape. If not, the sample was re-run and considered positive only with another positive result recorded.

We built general linear models to assess which variables were important in explaining the probability of infection for individuals, with one model being constructed for each of the two focal pathogens (i.e. one to explain infection probability with *Bd*, one to explain infection probability with *Rv*). Each model included intrinsic and extrinsic covariates known or predicted to have an effect on host pathogen dynamics, either directly or indirectly. The covariates of interest were the species and life-stage of the sampled individual, site (Ercina or Lloroza) and season (spring = March-June; summer = July–September). Finally, to ascertain whether infection with one of these pathogens was associated with infection with the other, for each of our two models, we included infection status with the other pathogen as a predictor of infection with the focal pathogen (e.g. we included *Bd* infection status as a predictor variable in the model seeking to explain probability of infection with *Rv*, and vice versa). The five covariates included were therefore: species (2 levels); location (2 levels); season (2 levels); life-stage (2 levels); and *Bd*/*Rv* infection status (2 levels). These variables have 31 possible combinations including three-way interactions, which were considered in building two explanatory models for *Rv* and *Bd* infection status. We used forward stepwise nominal regressions with the minimum AICc as stopping rule.

As previous data suggested that site and species are important factors in the infection status with both of these pathogens, we also conducted more focused analyses looking at whether the patterns of uninfected, singly infected with either pathogen, and co-infected individuals were consistent with those we would expect by chance. To do this we split the dataset and analysed data from Ercina for infection patterns in *Ao*, and data from Lloroza for infection patterns in *Ia*. We ran Pearson's chi-squared tests to determine whether the observed patterns of infection were evenly distributed, or whether there was an association between prevalence of infection with *Bd* and *Rv*. These combinations of sites and species were chosen as previous analyses suggested that those species represent the most important hosts in those sites (Bielby et al*.* in review). To test the sensitivity of our results we also conducted analyses of the same type but instead had two separate categories of singly infected individuals, in which data were separated depending on whether they were infected with *Bd* alone and *Rv* alone.

To determine whether intensity of pathogen infection levels were correlated with one another, infection data were log-transformed and analysed using Pearson’s correlation. All statistical analyses were performed with JMP Pro 14 (SAS Inc.).

All research was performed in accordance with relevant guidelines and regulations and under licence from the Parque Nacional Picos de Europa. All experimental protocols were approved by Comisión Ética de Experimentación Animal MNCN-CSIC (666/2018).

## Supplementary information

Supplementary Informaion.

## Data Availability

The datasets analysed during the current study are available from the corresponding author on reasonable request.
