# Supplementary material for: Single infection with Batrachochytrium dendrobatidis or Ranavirus does not increase probability of co-infection in a montane community of amphibians
Source: Sci Rep. 2020 Dec 3;10:21115. doi: 10.1038/s41598-020-78196-3 (PMC7712875; doi:10.1038/s41598-020-78196-3)

Single infection with *Batrachochytrium dendrobatidis* or *Ranavirus* does not increase probability of co-infection in a montane community of amphibians

Jaime Bosch[1,2]*, Camino Monsalve-Carcaño[2], Stephen J. Price[3,4] & Jon Bielby[5]

[1]Research Unit of Biodiversity (CSIC, UO, PA), Oviedo University - Campus Mieres, Spain. [2]Museo Nacional de Ciencias Naturales-CSIC, José Gutiérrez Abascal 2, 28006 Madrid, Spain. [3]UCL Genetics Institute, Darwin Building, Gower Street, London WC1E 6BT. [4]Institute of Zoology, Zoological Society of London, Regents Park, London NW1 4RY. [5]Liverpool John Moores University, School of Natural Sciences and Psychology, James Parsons Building, Byrom Street, Liverpool, L3 3AF

Supplementary Table S1. Step history reports for the explanatory models for *Bd* and Rv infection status showing, as each step is taken, the effect of adding a term to the model. The minimum corrected Akaike Information Criterion (AICc) was used as the stopping rule to choose the best models. The plot criterion history shows the AICc and BIC (minimum Bayesian Information Criterion) versus the number of parameters for the explanatory models for *Bd* and Rv infection status.

Batrachochytrium dendrobatidis

Step History

Step		Parameter	Action	L-R ChiSquare	"Sig Prob"	Entry ChiSquare	Entry "Sig Prob"	RSquare	p	AICc	BIC
1		species{Aobs-Ialp}	Entered	108.7313	0.0000	109.358	1.4e-25	0.2895	2	270.92	278.341
2		season{spring-summer}	Entered	13.11063	0.0003	13.0159	0.00031	0.3244	3	259.849	270.96
3		species{Aobs-Ialp}*locality{Ercina-Lloroza}	Entered	14.70278	0.0001	10.8125	0.00449	0.3635	5	249.266	267.718
4		Rv inf status{1-0}	Entered	9.277501	0.0023	8.71158	0.00316	0.3882	6	242.069	264.17
5		locality{Ercina-Lloroza}*live stage{larvae-postmet}	Entered	6.275367	0.0122	7.48654	0.02368	0.4049	8	239.996	269.355
6		species{Aobs-Ialp}*season{spring-summer}*live stage{larvae-postmet}	Entered	7.258281	0.0071	10.3361	0.03513	0.4243	12	241.314	285.017
7		Rv inf status{1-0}*species{Aobs-Ialp}*locality{Ercina-Lloroza}	Entered	9.899232	0.0017	9.6894	0.0214	0.4506	15	238.001	292.308
8		Rv inf status{1-0}*live stage{larvae-postmet}	Entered	1.481195	0.2236	1.43485	0.23097	0.4546	16	238.745	296.557
9		Rv inf status{1-0}*species{Aobs-Ialp}*season{spring-summer}	Entered	3.580706	0.0585	2.99967	0.22317	0.4641	18	239.662	304.437
10		locality{Ercina-Lloroza}*season{spring-summer}	Entered	0.577505	0.4473	0.31475	0.57478	0.4656	19	241.356	309.589
11		Rv inf status{1-0}*species{Aobs-Ialp}*live stage{larvae-postmet}	Entered	1.441e-8	0.9999	1.1e-18	1	0.4656	20	243.644	315.32
12		Best	Specific	.	.	1.1e-18	1	0.4506	15	238.001	292.308

Criterion History

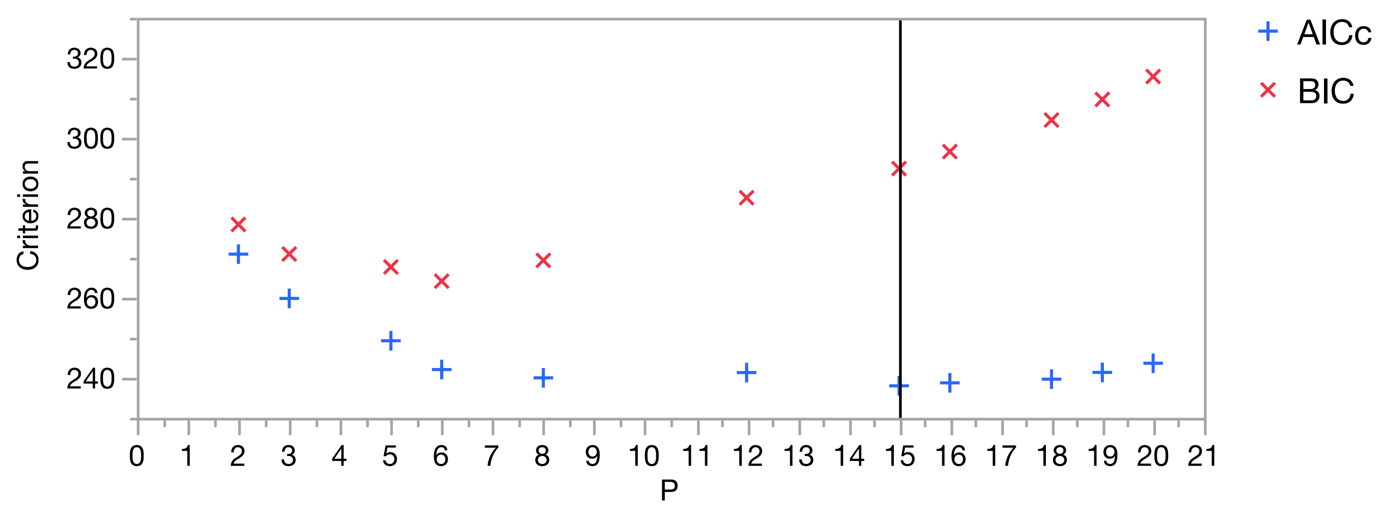

Ranavirus

Step History

Step		Parameter	Action	L-R ChiSquare	"Sig Prob"	Entry ChiSquare	Entry "Sig Prob"	RSquare	p	AICc	BIC
1		locality{Lloroza-Ercina}*season{summer-spring}	Entered	115.9224	0.0000	56.8766	2.7e-12	0.2716	4	319.071	333.86
2		Bd inf status{1-0}	Entered	9.70954	0.0018	9.74892	0.00179	0.2943	5	311.429	329.88
3		live stage{larvae-postmet}	Entered	11.17142	0.0008	11.0936	0.00087	0.3205	6	302.338	324.439
4		Bd inf status{1-0}*species{Ialp-Aobs}	Entered	4.450289	0.0349	3.8002	0.14955	0.3309	8	302.09	331.449
5		Bd inf status{1-0}*species{Ialp-Aobs}*locality{Lloroza-Ercina}	Entered	9.386233	0.0022	7.4748	0.05821	0.3529	11	299.114	339.253
6		locality{Lloroza-Ercina}*live stage{larvae-postmet}	Entered	4.429762	0.0353	7.22635	0.00718	0.3633	12	296.85	340.553
7		species{Ialp-Aobs}*season{summer-spring}	Entered	1.677108	0.1953	1.91484	0.16643	0.3672	13	297.353	344.606
8		Bd inf status{1-0}*live stage{larvae-postmet}	Entered	0.787285	0.3749	0.79268	0.37329	0.3691	14	298.761	349.549
9		season{summer-spring}*live stage{larvae-postmet}	Entered	0.694123	0.4048	0.66798	0.41376	0.3707	15	300.277	354.585
10		Bd inf status{1-0}*season{summer-spring}	Entered	3.261989	0.0709	2.54395	0.11072	0.3783	16	299.241	357.053
11		locality{Lloroza-Ercina}*season{summer-spring}*live stage{larvae-postmet}	Entered	9.038e-8	0.9998	1.2e-10	0.99999	0.3783	17	301.482	362.783
12		Bd inf status{1-0}*species{Ialp-Aobs}*season{summer-spring}	Entered	2.754e-8	0.9999	1.4e-16	1	0.3783	18	303.738	368.513
13		species{Ialp-Aobs}*live stage{larvae-postmet}	Entered	2.87e-8	0.9999	1.3e-15	1	0.3783	19	306.011	374.244
14		Bd inf status{1-0}*species{Ialp-Aobs}*live stage{larvae-postmet}	Entered	1.842e-7	0.9997	2.3e-10	0.99999	0.3783	20	308.298	379.974
15		Bd inf status{1-0}*season{summer-spring}*live stage{larvae-postmet}	Entered	1.695e-9	1.0000	1.8e-19	1	0.3783	21	310.602	385.704
16		Best	Specific	.	.	1.8e-19	1	0.3633	12	296.85	340.553

Criterion History

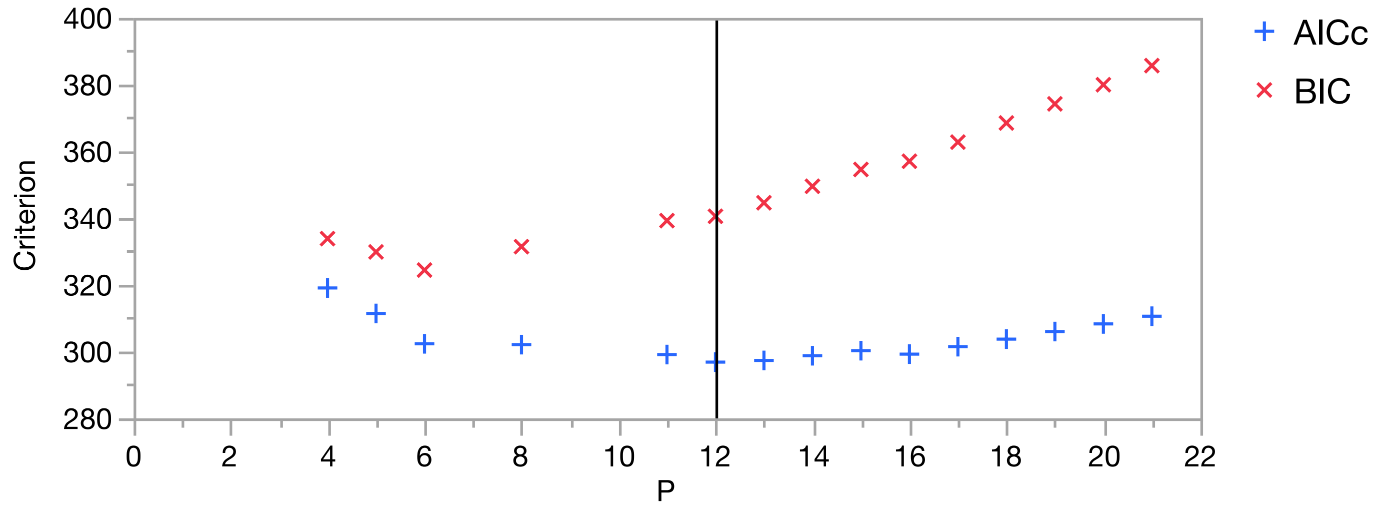

Supplement: Supplementary file 1 — Supplementary Informaion. [file 41598_2020_78196_MOESM1_ESM.pdf]
